# Ecophysiology, genotoxicity, histopathology, and gene responses of naphthalene injected *Colossoma macropomum* (Cuvier, 1818) exposed to hypoxia

**DOI:** 10.1590/1678-4685-GMB-2018-0084

**Published:** 2019-06-13

**Authors:** Samara Silva de Souza, Grazyelle Sebrenski da Silva, Vera Maria Fonseca de Almeida-Val

**Affiliations:** 1 Laboratory of Ecophysiology and Molecular Evolution, National Institute for Research in the Amazon (INPA), Manaus, AM, Brazil; 2 Institute of Biological Science (ICB), Universidade Federal do Amazonas (UFAM), Manaus, AM, Brazil

**Keywords:** Naphthalene, hypoxia, tumor suppressor gene *tp53*, DNA damage, tambaqui

## Abstract

The present study aimed to evaluate the biological responses of *Colossoma macropomum* to naphthalene injection and subsequent hypoxia exposure, emphasizing the expression of the tumor suppressor gene *tp53.* Tambaquis were intraperitoneally injected with naphthalene (50 mg/kg) and, after 96 hours, the fish were transferred to respirometry chambers and, submitted to progressive hypoxia for the determination of critical PO_2_. In a subsequent experiment, the fish received an intraperitoneal injection of naphthalene and were kept for 96 hours under normoxia. Successively, fish were challenged with acute hypoxia (PO_2_<PO_2_ crit) during 6 hours. We observed that the PO_2_ crit was not affected by naphthalene injection. Moreover, hematological parameters were modulated only in response to hypoxia. Fish with naphthalene injection plus hypoxia exposure presented altered activity of the GST and CAT enzymes. Exposure to naphthalene also resulted in DNA damages, which was not influenced by hypoxia. Hypoxia accentuated the hepatic lesions caused by naphthalene, as well as it also impaired the transcription of *tp53* in naphtalene injected fish, demonstrating the risks of contaminating aquatic environments, especially environments where hypoxic conditions are common and occur on a daily or on seasonal basis, as in the Amazon basin.

## Introduction

Several sources of aquatic pollution, including the presence of highly toxic polycyclic aromatic hydrocarbons (PAHs) affect aquatic organisms (Palanikumar *et al.*, 2013; Kochhan *et al.*, 2015; Sadauskas-Henrique *et al.*, 2017). Among PAHs, naphthalene stands out as one of the main constituents of crude oil (Negreiros *et al.*, 2011; Gusmão *et al.*, 2012; Omar-Ali *et al.*, 2015). Naphthalene toxicity is the result of the biotransformation process, which consists in cellular chemical reactions with the aim of making the compounds more soluble in water, facilitating their excretion. During biotransformation, besides the generation of more toxic intermediate compounds, the formation of reactive oxygen species (ROS) can occur. ROS are unstable molecules capable of inducing oxidative stress (Van der Oost *et al.*, 2003; Shi *et al.*, 2005) and several damages to the animal cells. ROS can interact with biological macromolecules inducing damage to proteins, lipids, and DNA (Van der Oost *et al.*, 2003). Several studies demonstrated that the presence of naphthalene and other PAHs can result in increases in the activity of antioxidant enzymes involved in the neutralization of ROS, as well as increased DNA damages and lipid peroxidation in the gills and liver of fish (Shi *et al.*, 2005; Palanikumar *et al.*, 2013; Sadauskas-Henrique *et al.*, 2017).

Damages caused by ROS can compromise the function of different tissues. Kochhann *et al.* (2015) observed the occurrence of gill damage in *Colossoma macropomum* exposed to crude oil. Such damages promote an increase in the diffusion barrier to reduce the contact between tissue and pollutant; however, they compromise tissue function, including oxygen uptake. Silva *et al.* (2017) demonstrated that the intraperitoneal injection of different benzo[a]pyrene (BaP) concentrations in *C. macropomum* resulted in the occurrence of irreparable histopathological damages in the liver, which may be directly related to the damages caused by ROS produced during the biotransformation of this PAH. The histopathological damages resulting from exposure to BaP reflect on the molecular responses of the species, since the exposure of those fish to BaP also affected the expression of genes related to the development of cancer, such as the oncogene *ras* and the gene *hif-1*α (Silva *et al.*, 2017).

In the Amazon basin, the effects of contamination by PAHs, such as naphthalene, can be more drastic, since its aquatic environments have particular characteristics, including seasonal and daily variations in the concentration of dissolved oxygen in water (Junk *et al.*, 1983; Val and Almeida-Val, 1995). Several studies have evaluated the effects of PAHs and hypoxia independently. However, the interaction between the two factors has been little explored in freshwater organisms. Fish studies demonstrated that exposure to hypoxia accentuates the negative effects of stressors such as ultraviolet radiation and crude oil, affecting the detoxification process and antioxidant defenses, as well as accentuating DNA and histopathological damages (Negreiros *et al.*, 2011; Dasgupta *et al.*, 2016; Groff *et al.*, 2010; Silva *et al.*, 2019). The combination of hypoxia and contaminants also affects the expression of genes involved in carcinogenesis. Silva *et al.* (2019) demonstrated that exposure of *C. macropomum* to Roundup^®^ herbicide and subsequent exposure to hypoxia resulted in down-regulation of *ras* oncogene expression.

Hypoxia plays an essential role in cell proliferation, angiogenesis, tumor progression, and metastasis. Hypoxia is toxic to cells. However, cancer cells survive and proliferate in a hypoxic environment, which contributes to the malignant phenotype and aggressive tumor behavior (Vaupel and Mayer, 2007; Lee *et al.*, 2009; Eales *et al.*, 2016). Both factors naphthalene and hypoxia, can modulate the expression of genes involved in cell cycle control and DNA repair, such as the tumor suppressor gene *tp53*, once this gene expression is influenced by the occurrence of DNA damage (Kastan *et al.*, 1991; Park *et al.*, 2006). As a transcription factor, the p53 protein, encoded by the *tp53* gene, is critical in the maintenance of genomic integrity. The p53 protein is also involved in the restriction of neoplastic progression in mammals (Donehower *et al.*, 1992) and fish (Berghmans *et al.*, 2005; Tu *et al.*, 2016). Studies report that fish captured at contaminated sites or exposed to xenobiotics show increased levels of *tp53* mRNA (Mai *et al.*, 2012; Ruiz *et al.*, 2012; Williams and Hubberstey, 2014). Hypoxia also regulates *tp53*. Most studies with *tp53* are performed using mammalian cells *in vitro* (Koumenis *et al.*, 2001; Hammond *et al.*, 2002; Cosse *et al.*, 2009; Chen *et al.*, 2010). *In vivo*, hypoxia may either increase or decrease the *tp53* transcription in the white shrimp (*Litopenaeus vannamei*) depending on the tissue type (Felix-Portillo *et al.*, 2016; Nuñez-Hernandez *et al.*, 2018).

Fish are regularly exposed to several contaminants in the aquatic environment, making them more susceptible to damage caused by pollutants. In the Amazon basin, *Colossoma macropomum* has been widely used as a bioindicator in toxicological studies of aquatic contamination by crude oil and petroleum derivated products (Duarte *et al.*, 2010; Kochhann *et al.*, 2013, 2015; Sadauskas-Henrique *et al.*, 2016, 2017; Silva *et al.*, 2017). These preliminary studies demonstrate that tambaqui is a good model for ecotoxicological studies, including for analysis of gene expression related to the development of cancer, since it is able to resist to different environmental contaminants, as well as to the combination between contaminants and changes in water quality, such as variations in dissolved oxygen concentrations, a common factor in Amazonian aquatic environments (Val and Almeida-Val, 1995; Kochhann *et al.*, 2013, 2015; Sadauskas-Henrique *et al.*, 2016, 2017; Silva *et al.*, 2017; Silva *et al.*, 2019). Analysis of *tp53* gene expression combined with antioxidant responses and damage resulting from exposure to naphthalene in a species with adaptations that allow them to survive in hypoxic environments can provide information on molecular mechanisms that allow survival of the species under stress conditions. Thus, this work aimed to investigate the tumor suppressor *tp53* gene expression after naphthalene injection and subsequent exposure to acute hypoxia. To verify the oxidative stress and damages, we analyzed changes in antioxidant and biotransformation enzymes, as well as DNA and histopathological damages in the liver of *C. macropomum*.

## Material and Methods

### Experimental fish

Tambaqui juveniles (*Colossoma macropomum*) were purchased from a local fish farm (Fazenda Santo Antônio: 02º44’802’’S; 059º28’836’’W, Amazonas, Brazil) and transferred to the Laboratory of Ecophysiology and Molecular Evolution at INPA (National Institute for Research in the Amazon, Manaus, Amazonas). Animals were firstly acclimated outdoors for 60 days in 3000-L polyethylene aerated tanks with constant water circulation. During the acclimation period, fish were fed daily until satiation with commercial pelleted food (36% protein, Nutripeixe-Purina). Feeding was suspended 24 hours before the beginning of the experiments. All experimental procedures followed CONCEA Brazilian Guidelines for Animals Use and Care, under INPAs authorization by the Committee of Ethics for Use of Animals (CEUA protocol number 043/2015). All fish used in the present study were sexually immature.

### Experiment 1: Determination of critical oxygen tension

The first experiment was performed only to determine the critical oxygen tension (PO_2_ crit) and define the oxygen concentration to be used in the experiments under hypoxic conditions (Experiment 2). The PO_2_ crit is the partial oxygen tension below which the availability of oxygen becomes insufficient for the regulation of the metabolic rate of the organism and, the animal starts to conform to the tension of oxygen, modifying its respiration rate according to the environmental PO_2_ (Pörtner *et al.*, 1985). Five individuals per treatment, with 27.9 ± 6.8 g body mass and 10.9 ± 1.2 cm length (mean ± standard deviation), were acclimated for 48 hours before the start of the experiments. They were divided into three treatments and individually placed in 3 L glass aquaria. In the first treatment, the fish did not receive the injection (group without injection - GWI). In the second treatment (Sham) fish were injected with the vehicle solution (corn oil Sigma^®^). In the third treatment (Naph) fish were injected with the solution containing naphthalene dissolved in corn oil. Naphthalene was administered to fish via intraperitoneal (i.p) injection based on the body weight of each fish. Before injections, all animals were anesthetized in water at 10 ºC for 30 s and were then weighed and measured. The individuals of the Naph group all received the same dosage (50 mg/kg). This naphthalene concentration used in the present study was determined from other studies where the authors used the same route of administration (Tintos *et al.*, 2007; Gesto *et al.*, 2009).

Fish were returned to their aquarium after the injection, and after 96 hours they were individually transferred to respirometry chambers (1.7 L) inside a bath aquarium and maintained for 1 hour in a recirculating system with continuous water flush. The control of the recirculation cycle was done by AutoResp software (Loligo System). The amount of oxygen in the chambers was measured using OXY-4 and Witrox-4 (Loligo System) oximeters. Then, the flow phase was stopped, and the fish were exposed to progressive hypoxia so that the decrease in PO_2_ occurred as the oxygen available inside the chambers was being consumed. Subsequently, the oxygen consumption rates were calculated, and PO_2_ crit was determined using the SegReg program (www.waterlog.info). The methodology was the same as the one used by Campos *et al.* (2016).

### Experiment 2: Naphthalene contamination and subsequent exposure to hypoxia

Tambaqui juveniles (58.8 ± 6.3 g and 14.0 ± 0.5 cm) were divided into three treatments, six individuals for each treatment, and separated into glass aquaria where they were maintained for 48 hours before the beginning of experiments. In the first treatment (group GWI), the fish received no intraperitoneal injection. In the second treatment (group Sham), the fish received corn oil, and in the third treatment, the fish received naphthalene dissolved in corn oil (group Naph). Fish from group Naph were injected intraperitoneally with 50 mg/kg of naphthalene using corn oil as vehicle. The injections occurred as described above. After injections, fish were returned to their glass aquaria and kept under constant temperature, aeration, and pH (temperature = 27.04 ºC ± 0.79; oxygen = 6.0 ± 0.25 mg O_2_.L^-1^; pH= 7.2 ± 0.55 - mean ± standard deviation) for 96 hours. After 96 hours, the PO_2_ for fish in the hypoxia groups was decreased slowly by pumping N_2_ gas directly into the water, and the animals were kept for six hours under acute hypoxia, at 1.08 ± 0.10 mg O_2_.L^-1^. The normoxia groups were kept in the respective aquaria for the same six hours under constant aeration at 6.07 ± 0.25 mg O_2_.L^-1^. At the end of each experiment, blood samples were immediately drawn from the caudal vein into heparinized syringes. Blood was used in the hematological analysis and comet assay. After blood sampling, fish euthanasia occurred by a concussion to their heads, followed by an immediate cut in the spinal cord. Then, the liver was excised and stored at -80 ºC until analysis. Liver was used in the analysis of the activity of GST and CAT, LPO, histopathological damage, and gene expression.

### Hematological assays

For hematocrit determination (Hct) blood samples were transferred to microhematocrit capillaries and centrifuged at 12,000 rpm (Centrifuge 3400, FANEM) for 10 minutes. Reading of the percentage of sedimentation (%) was performed using a standard scale (Goldenfarb *et al.*, 1971). Hemoglobin ([Hb]) concentration was determined spectrophotometrically at 540 nm according to the cyanmethemoglobin method (Kampen and Zijlstra, 1961). Red blood cell (RBC) counts were done in a Neubauer chamber. The corpuscular constants were calculated according to Brown (1976). Plasma was obtained after blood centrifugation at 604 g for 10 minutes for glucose analysis. Plasma glucose was determined using the Glucose Liquicolor Kit (InVitro^®^).

### Biochemical Analysis

Liver samples were homogenized in cold buffer solution (200 mM Tris-Base, 1 mM EDTA, 1 mM dithiothreitol, 500 mM sucrose, 150 mM KCL, pH 7.6) and centrifuged at 9,000 x g for 30 min at 4 ºC. The supernatant was used to analyze glutathione S-transferase (GST) and catalase (CAT) activity. To determine the extent of lipid peroxidation (LPO), liver samples were weighed, homogenized (1:2 w/v) in the same buffer as used for GST and CAT, and centrifuged at 1,062 x g for 10 min at 4 ºC. GST activity was determined as described by Keen *et al.* (1976) using CDNB (1-chloro-2,4-dinitrobenzene) as a substrate.Absorbance was measured spectrophotometrically at 340 nm, and GST activity was calculated as nmol CDNB conjugate/min/mg protein. The determination of CAT activity was done according to the methodology described by Beutler (1975), which consists of measuring the rate of degradation of hydrogen peroxide (H_2_O_2_) at 240 nm. CAT activity was expressed in μmol H_2_O_2_ min/mg protein. LPO in the liver was quantified spectrophotometrically at 560 nm by the Ferrous Oxidation/Xylenol Orange method, as described by Jiang *et al.* (1992). The LPO concentration was expressed in μM of CHP (Cumene hydroperoxide) / mg protein.

### Protein determination

Total protein in the liver extracts used for the enzyme analyses was measured spectrophotometrically at 595 nm according to the method described by Bradford (1976), using bovine serum albumin as standard.

### Comet assay

**Figure 1:**
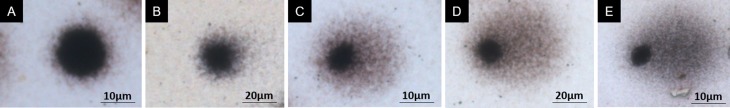
Class of DNA damages (comet assay) observed in blood cells of *C. macropomum* in the three experimental groups: Group Without Injection (GWI), Group with corn oil injection (Sham); and Group with naphthalene injection (Naph) after 96 h normoxia exposure followed by 6 h of hypoxia. Class 0, without damages (A), class 1 (B), class 2 (C), class 3 (D), and class 4, maximum damages (E). The images were captured with 400 x magnication.

The comet assay was performed in alkaline conditions, as described by Singh *et al.* (1988) for lymphocytes and modified by Silva *et al.* (2000) for peripheral blood cells. Diluted blood with cell suspension was mixed with low melting point agarose 0.75% (Gibco BRL) and spread on the slides pre-covered with normal melting agarose (1.5%) prepared in phosphate-buffered saline (PBS). The slides were placed in a cold lysing solution. After fixation, the slides were placed in an electrophoresis chamber and submerged in freshly prepared alkaline buffer (pH 13.0) for 20 min before; a current 25 V was applied for 15 min. Subsequently, the slides were washed with 0.4 M Tris buffer (pH 7.5) and stained with silver solution for 15 min at 37 °C. Using an optical microscope (Leica DM205) at 100 x magnification, 100 cells were analyzed from each of two replicate slides randomly selected from each fish. The damage was visually classified into five classes according to tail size. Counting was performed observing the tail size formed due to migration of damaged DNA after electrophoresis. Class 0 corresponds to intact DNA, without tail; class 1, low damage index; class 2, intermediate damage; class 3, high damage; and class 4, extreme damage. Figure 1 illustrates the different damage classes that occurred in the present work. The genetic damage index (GDI) for each fish was calculated as the number of cells observed in each damage class multiplied by the value of the class damage according to the following formula:

DGI=(Ax0)+(Bx1)+(Cx2)+(Dx3)+(Ex4)

(Kobayashi *et al.*, 1995), where: A= total cells without damage; B= total cells with damages class 1; C= total cells with damages class 2; D= total cells damges class 3; and E= total cells damages class 4.

### Liver histopathological analysis

Liver samples (n=6) were fixed in ALFAC solution (80% ethanol, 5% glacial acetic acid, and 4% formaldehyde) for 15 hours and transferred to 70% ethanol. Subsequently, tissues were dehydrated in successive ethanol baths, diaphanized in xylol, and included in paraffin. Blocks were sliced in 5 μm thick sections, stained with hematoxylin-eosin (HE) and read by light microscopy (Leica DM2015) at 40x. Two sections of liver lesions were qualitatively analyzed for each fish using the levels classified by Poleksic and Mitrovic-Tutundic (1994), modified by Silva (2004). The carbohydrate stainning histochemistry was applied for the evaluation of hepatic glycogen, using the PAS method (Periodic Acid Schiff).

### Total RNA extraction and first-strand (cDNA) synthesis

Total RNA was extracted from liver (n = 4, for each treatment) using Trizol Reagent (InvitrogenTM, Life Technologies). The concentration and purity of total RNA samples were checked in a NanoDrop^®^ 2000 Spectrophotometer (Thermo Scientific). RNA integrity was verified by an electrophoretic run in an agarose gel (2%). Possible residues of genomic DNA were removed by the DNase I kit (Invitrogen, Life Technologies). cDNA synthesis was performed using the Platus Transcriber RNase H cDNA First Strand kit (Sinpase Inc.) following the manufacturer’s instructions.

### Sequencing and primer obtention

Primers for the *tp53* gene (forward: ^5’^GGAGTGGC TGATTCAGAG^3’^; reverse: ^5’^TTAAGGAGAGCGGTC ATG^3’^; efficience: 100.24%; R^2^: 0.98) were designed from sequences obtained from the tambaqui transcriptome (Prado-Lima and Val, 2016) (Access number: SRP062336). Sequences of the *tp53* gene were validated using the BLASTn tool in NCBI (http://www.ncbi.nlm.nih.gov). Primers were designed in Oligo Explorer 1.1.2 software. For normalization of target gene expression, two housekeeping genes were used: ribosomal gene *28S* (forward: ^5’^CGGGTTCGTTTGCG TTAC^3’^; reverse: ^5’^AAAGGGTGTCGGGTTCAGAT^3’^; efficience: 98.19; R^2^:0.99) and transcription elongation factor *ef-1*α (forward: ^5’^GTTGGTGAGTTTGAGGCT GG^3’^; reverse: ^5’^CACTCCCAGGGTGAAAGC^3’^; efficience: 99.09; R^2^: 0.99). These genes were used in prior studies with both exposures to PAH and hypoxia, showing stable expression (Silva *et al.*, 2017; Silva *et al.*, 2019).

### Quantitative real-time (qPCR) analysis

qPCR reactions were performed in a Viia 7 Dx PCR-System (Applied Biosystem). Each reaction was performed in triplicate with 1000 ng cDNA using Fast SYBR^®^ Green PCR Master Mix (Applied Biosystems). The reaction condition was: heating for 2 min at 50 °C and 95 °C for 10 min, followed by 40 cycles of 95 °C for 15 s and 60 °C for 1 min (annealing temperature of all primers). Differences in gene expression were calculated using the method 2^-ΔΔCt^ (Livak and Schmittgen, 2001).

### Statistical analysis

Data are expressed as the mean ± standard error of the mean (mean ± SEM). Prior to comparative statistical analyses, data were assessed for normality and homogeneity of variance. A one-way analysis of variance (ANOVA) was applied to verify differences in the critical oxygen tension (PO_2_ crit). A two-way ANOVA was applied in the analysis of hematological parameters ([Hb], Hct, RBC, MCV, MCH, MCHC, and plasma glucose), GST and CAT activity, LPO, comet assay, and gene expression, using oxygen concentration and treatments as factors. Significant differences between the means were scored by the Tukey test, at the 5% level of significance. A principal component analysis (PCA) was performed to verify whether the clustering of the analyzed variables was determined mainly by the naphthalene injection or by the oxygen concentration. The statistical tests were run using SigmaStat 3.5 and Statistica 7.0 programs.

## Results

### Critical oxygen tension

No difference was detected in the critical oxygen tension (PO_2_ crit) for *C. macropomum* among treatments (*p*=0.825; F=0.195). The critical oxygen tensions were 1.56 ± 0.12, 1.66 ± 0.13 and 1.56 ± 0.14 mg O_2_.L^-1^ for the GWI, Sham, and Naph groups, respectively. The metabolic rate at reduced levels of dissolved oxygen in water decreased in the same way in fish from all three treatments, demonstrating that naphthalene did not affect the oxygen consumption of *C. macropomum*.

### Hematological parameters

Interestingly, no differences were detected in hematological parameters in fish of the three treatments (GWI; Sham; and Naph) at normoxia (Table 1). Conversely, hypoxia affected some hematological parameters in fish priorly exposed to GWI, Sham and Naph groups; hemoglobin concentration increased in the Sham and Naph groups in hypoxia compared to normoxia (*p* < 0.05; F = 16.261). There was an increase in hematocrit (*p* < 0.001; F = 84.712), in RBC (*p* < 0.05; F = 28.825), and there was a decrease in MCHC (*p* < 0.05; F = 25.870) in fish kept under hypoxia when compared to the same groups in normoxia. The lower oxygen concentration also influenced plasma glucose levels in fish of all treatments (*p* < 0.001; F = 57.404), resulting in the increase of this metabolite under hypoxic conditions compared to normoxia (*p* < 0.05), with no differences, however, as above mentioned among the three treatments in each condition: normoxia or hypoxia.

**Table 1:** Hematological parameters levels in *C. macropomum* in the groups without injection (GWI), with corn oil injection (Sham) and naphthalene injection (Naph) after 96 h to normoxia exposure followed by 6 h of hypoxia. *Indicates statistical difference between normoxia and hypoxia of the same treatment. Significance value *p* <0.05.

	GWI		Sham		Naph	
Parameter	Normoxia	Hypoxia	Normoxia	Hypoxia	Normoxia	Hypoxia
Hct (%)	21±0.43	28.7±1.16*	20.2±1.17	28±1.18*	21.6±1.01	27.8±0.49*
Hb (g/dL)	8.9±0.25	9.3±0.73	8.6±0.32	10.5±0.28*	8.9±0.28	10.2±0.12*
RBC (x10^6^/mm^3^)	1.5±0.09	1.7±0.04*	1.4±0.05	1.77±0.07*	1.5±0.09	1.8±0.04*
MCV (μm^-3^)	154±4.03	164.6±5.35	150.1±5.76	152.5±3.71	140.2±4.25	149.6±5.48
MCH (pg)	59.9±2.64	55±3.09	64.2±3.09	58.6±2.73	65.7±4.23	57.4±0.62
MCHC (%)	41.2±0.96	34.5±2.14*	44.0±1.18	36.9±0.93*	42.1±2.21	37.0±1.07*
Glucose (mg/dl)	49.8±4.57	84.1±9.27*	41.6±3.45	87.5±8.28*	43.4±6.20	96.4±9.08*

### Biochemical analysis

**Figure 2:**
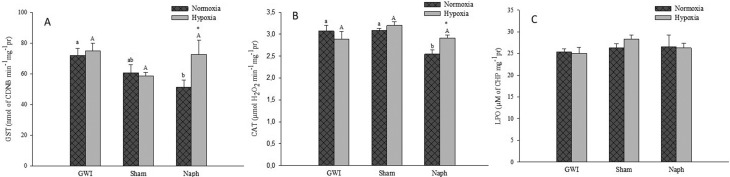
GST (A) and CAT (B) activity and lipoperoxidation (LPO) (C) in the liver of *C. macropomum* in the three experimental groups: Group Without Injection (GWI), Group with corn oil injection (Sham); and Group with naphthalene injection (Naph) after 96 h normoxia exposure followed by 6 h of hypoxia. Small caps letters (a and b) indicate differences among treatments in normoxia. Capital letters (A and B) indicate differences among treatments in hypoxia. *Indicates differences between normoxia and hypoxia at the same treatment. Significance level of the Tukey test was *p* <0.05.

Hepatic GST activity was influenced by the Naph injection (*p* = 0.038; F = 3.659), but there was no influence of hypoxia (*p* = 0.113; F = 2.675), or interaction between the factors (*p* = 0.112; F = 2.366). GST activity decreased 1.4 fold in fish injected with naphthalene (*p* <0.05) compared to the GWI in normoxia. There were no differences among the three treatments under hypoxia. However, fish that received Naph injection and were subsequently exposed to hypoxia showed a 1.4 fold increase in GST activity compared to those remaining under normoxia (*p* <0.05). There were no differences between GWI and Sham in both normoxia and hypoxia groups (Figure 2A).

Hepatic CAT activity was influenced by Naph (*p* = 0.002; F = 7.513) compared to GWI and Sham groups; however, there was no influence of hypoxia (*p* = 0.254; F = 1.351) or interaction between the factors (*p* = 0.052; F = 3.267). Enzyme activity decreased 1.2 fold in fish contaminated with Naph (*p* <0.05) compared to the GWI and Sham in normoxia. No differences between treatments were observed in fish groups under hypoxia. An increase in CAT activity occured in fish contaminated with naphthalene and later exposed to hypoxia in comparison to fish that remained in normoxia (*p* <0.05). There were no differences between GWI and Sham in both normoxia and hypoxia groups (Figure 2B). Moreover, lipid peroxidation in the liver presented no differences among all treatments in both normoxia and hypoxia fish groups (Figure 2C).

### Genotoxic damage

**Figure 3:**
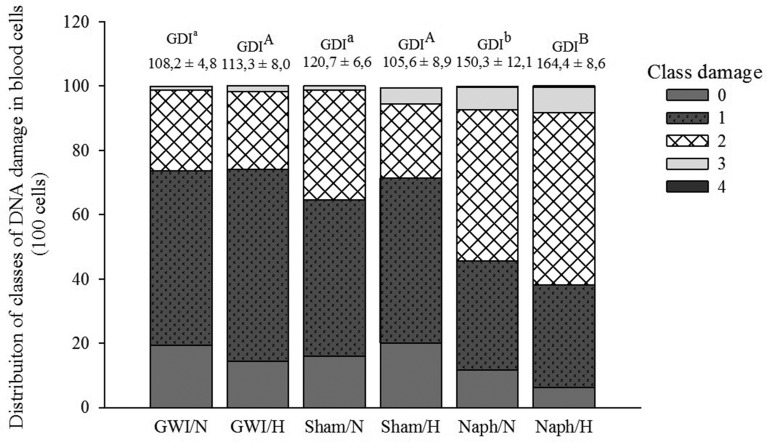
Distribution of classes of DNA damage in *C. macropomum* blood cells in the three experimental groups: Group Without Injection (GWI); Group with corn oil injection (Sham); and Group naphthalene injection (Naph) after 96 h normoxia exposure (N) followed by 6 h of hypoxia (H). The Genetic Damage Index (GDI) is identified in each treatment (on the bars). Small cap letters (a and b) indicate statistical difference among treatments in normoxia. Capital letters (A and B) indicate statistical difference among hypoxia treatments. The statistical significance value was *p* <0.05.

Injection with Naph resulted in the increase in the genetic damage index (GDI) in blood cells (*p* <0.05; F = 19.259). The exposure to hypoxia did not increase these damages (*p* = 0.845, F = 0.0388) and there was no interaction between the factors (*p* = 0.228; F = 1.556). Increases in DNA damage of 1.4 and 1.25 fold (*p*<0.05) were observed in normoxic *C. macropomum* treated with Naph compared to the GWI and Sham groups, respectively. In hypoxic fish injected with Naph, the increase in DNA damage was 1.45 fold higher (*p* <0.001) compared to GWI and Sham. No difference between normoxic and hypoxic fish groups was observed (Figure 3). With regard to DNA damage levels in blood cells, fish of the GWI and Sham groups, both in normoxia and hypoxia, presented a prevalence of damages class 1, where, out of the 100 cells analyzed for GWI, 54.2 and 59.8% were characterized as class 1 in normoxia and hypoxia, respectively. For the Sham group, class 1 appeared in 48.7, and 51.4% of cells in fish kept both under normoxia and hypoxia, respectively. The DNA damage class 2 was predominant in fish injected with Naph, being 47,2% for fish kept in normoxia and 53.5% after hypoxia exposure. DNA damage class 3 occurred in 7% of fish cells contaminated with Naph in the groups maintained in normoxia and hypoxia (Figure 3).

### Liver histopathology

**Figure 4:**
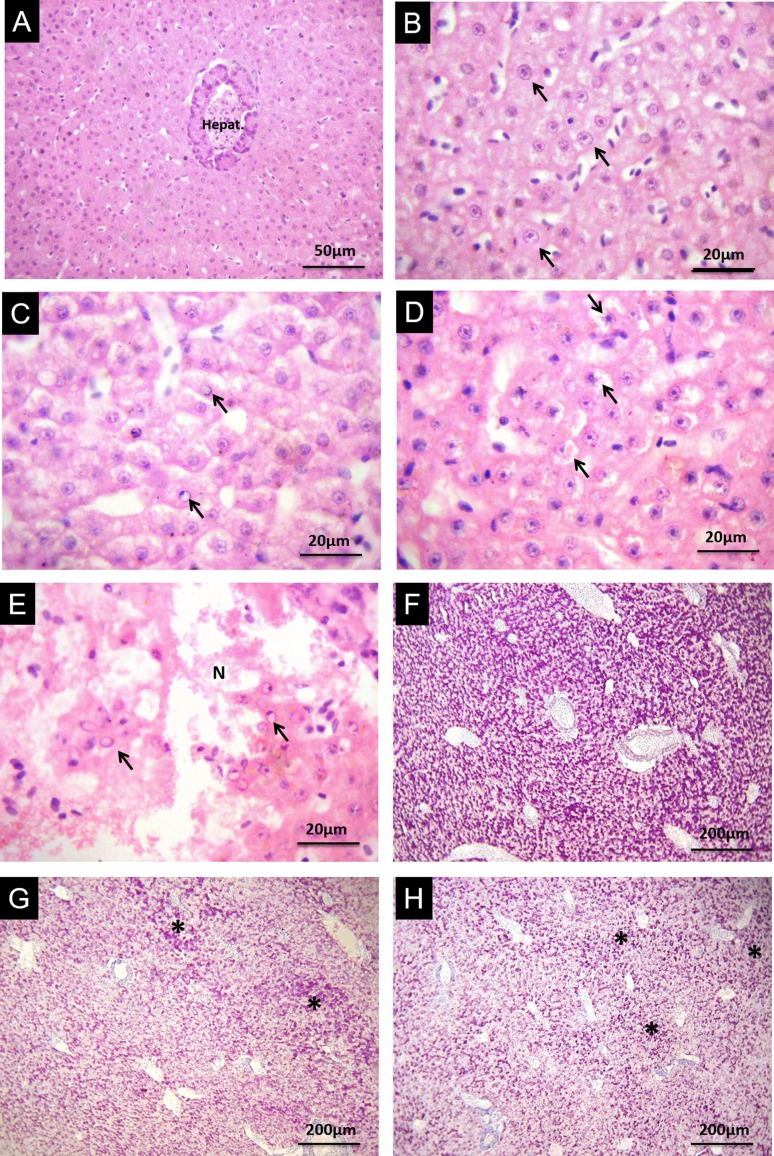
Liver histopathology of *C. macropomum* in the three experimental groups: Group Without Injection (GWI); Group with corn oil injection (Sham); and Group with naphthalene injection (Naph) after 96 h normoxia exposure followed by 6 h of hypoxia. (A) normal *C. macropomum* liver standing out the hepatopancreas. (B, C) after Naph injection in normoxia. Arrows indicate nuclei hypertrophy (B) and nuclei vacuolization (C). (D, E) after Naph injection and subsequent hypoxia exposure. Arrows indicate hepatocytes with nuclei degeneration (D) and nuclei vacuolization (E); N shows focal necrosis. Hematoxylin and eosin stain. (F) *C. macropomum* liver of the Sham group in normoxia demonstrating a strong positive reaction to PAS. (G, H) *C. macropomum* liver after hypoxia exposure: GWI (G) and Naph groups (H) demonstrating a weak reaction to PAS. Asterisks indicate areas with higher glycogen concentration. PAS corresponds to Periodic Acid Schiff Stain.

Healthy *C. macropomum* liver, similarly to other fish species, presented a parenchyma consisting of well delimited polyhedral hepatocytes, organized in linear cords surrounded by sinusoid capillaries as observed in the group without injection (GWI) (Figure 4A). In relation to qualitative analysis, most of the hepatic alterations noted in all treatments presented frequency ranging from rare to low, classified as mild or punctually localized (Table 2). Among stage I, nuclear hypertrophy ranged from frequent to highly frequent in fish treated with Naph under normoxic conditions (Figure 4B) and, in all hypoxia treatments. Cell deformation was a frequent change only in fish injected with Naph in normoxia. Nuclear vacuolation, classified as stage II damage, ranged from frequent to highly frequent in the Naph group in normoxia (Figure 4C) and all treatments in hypoxia (Figure 4E). Nuclear degeneration showed low frequency in fish treated with Naph under hypoxic conditions (Figure 4D). Necrotic foci were rare or had a low frequency in fish of all treatments, except for fish injected with naphthalene and subsequently exposed to hypoxia, where focal necrosis was a common lesion (Figure 4E).

**Table 2:** Qualitative distribution of histopathological damage and occurrence intensity (0 absent, 0+ rarely present, + low frequency, ++ frequent and +++ high frequency) on the liver of *C. macropomum* in the groups without injection (GWI), with corn oil injection (Sham) and naphthalene injection (Naph) after 96 h normoxia exposure followed by 6 h of hypoxia.

Lesion Type	Normoxia				Hypoxia		
	Stage	GWI	Sham	Naph	GWI	Sham	Naph
Nuclei hypertrophy	I	++	0+	++	+++	++	++
Cell hypertrophy	I	0+	0+	0+	0+	0+	0+
Nuclei in cell periphery	I	++	++	++	++	++	++
Cytoplasm vacuolization	I	0+	0+	0+	0+	++	++
Leukocyte infiltration	I	0+	0	0+	0+	0+	0+
Sinusoid dilation	I	++	+++	+++	+++	+++	+++
Cellular deformation	I	++	++	++	++	++	++
Derangement of hepatic cords	I	0	0	0+	0	0	0
Vessel congestion	II	++	++	++	++	++	++
Nuclei vacuolization	II	0+	0+	+++	+++	++	++
Nuclei degeneration	II	0+	0+	0+	0+	0+	++
Cytoplasm degeneration	II	++	++	++	++	++	++
Pyknotic nuclei	II	0+	0+	0+	0+	0+	0+
Cell disruption	II	0+	++	++	++	++	++
Focal necrosis	III	0+	++	++	++	0+	++

Hepatocytes of *C. macropomum* kept under normoxia and hypoxia reacted differently to PAS. Under normal oxygen conditions, hepatocytes from the GWI and Sham showed a strong positive reaction to PAS (Figure 4F), which demonstrates a vast reserve of glycogen. In the group injected with Naph, this response ranged from moderate to strong. Hepatocytes of fish kept under hypoxia showed a decrease in glycogen in all three treatments (Figure 4G, H), represented by a reaction that ranged from mild to moderate when compared to fish kept under normoxia.

### *tp53* expression

**Figure 5:**
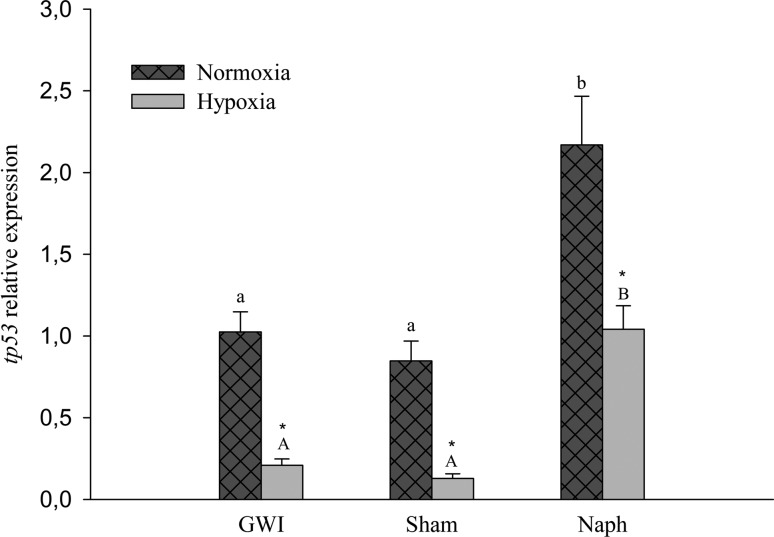
Relative *tp53* gene expression in *C. macropomum* liver in the the three experimental groups: Group Without Injection (GWI); Group with corn oil injection (Sham); and Group with naphthalene injection (Naph) after 96 h normoxia exposure followed by 6 h of hypoxia. Small cap letters (a and b) indicate differences among treatments in normoxia. Capital letters (A and B) indicate differences among treatments in hypoxia. *Indicates differences between normoxia and hypoxia of the same treatment (*p* <0.05).

Gene expression was influenced by the combination of low oxygen concentration (*p* <0.001; F = 53.935) and Naph injection (*p* < 0.001; F=34.470) (Figure 5). There was no interaction between factors (*p* = 0.369; F = 1.047). In fish contaminated with Naph, there was an increase in *tp53* mRNA levels in both normoxia and hypoxia compared to the GWI and Sham groups (*p* <0.001). In normoxia, *tp53* expression increased nearly 2.0 and 2.5 fold in fish after Naph injection compared to GWI and Sham, respectively. In fish exposed to hypoxia after contamination, *tp53* mRNA levels were about 5 fold higher than in the GWI group and 8 fold higher than in the Sham group. There were no differences in *tp53* mRNA levels between GWI and Sham in both normoxia and hypoxia groups. In fish exposed to hypoxia, gene expression was down-regulated compared to fish kept under normoxia (*p* <0.001). However, fish contaminated with Naph and subsequently submitted to hypoxia maintained high mRNA levels when compared to groups without injection and injected with corn oil (GWI, and Sham), respectively (Figure 5).

### Multivariate analysis

**Figure 6:**
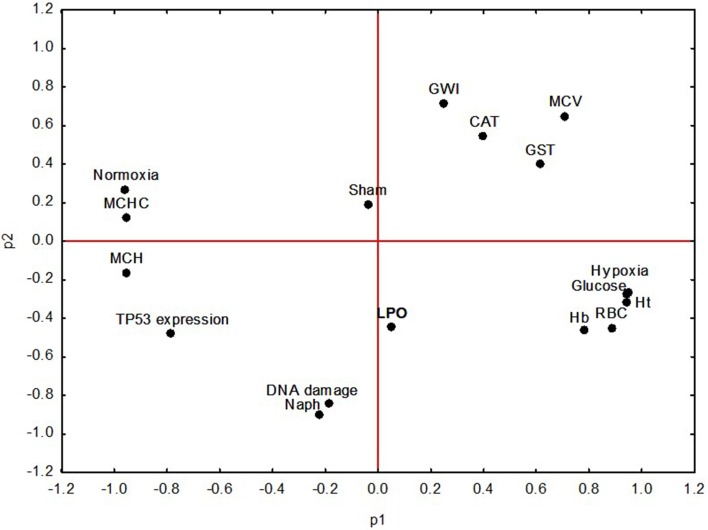
Biplot representing the distribution of PCA values for the variables analyzed in *C. macropomum* in the three experimental groups: Group Without Injection (GWI); Group with corn oil injection (Sham); and Group with naphthalene injection (Naph) after 96 h normoxia exposure followed by 6 h of hypoxia. All groups are compared and variation among variables is explained by p1 = 50.0% and p2 = 24.0%.

Principal component analysis (PCA) shows that the clustering of the different variables was mainly determined by oxygen concentration (normoxia and hypoxia) (Figure 6). The components p1 and p2 explained 74% of the original data variance (p1 = 50% and p2 = 24%). Normoxia and hypoxia were clustered in opposite quadrants as well as the GWI and Sham were clustered in the quadrant opposite to the Naph group. Variables are distributed in four clusters (Figure 6). One of the groups demonstrated the influence of hypoxia, represented by the clustering of variables Hb, Hct, glucose and RBC, which increased in fish kept in hypoxia, whereas, normoxia explains the values of MCHC, MCH and *tp53* expression. The group without injection (GWI) explained the GST, CAT and MCV variables. Higher Naph influence occurred on the DNA damage index, LPO and, to a lesser extent, on the *tp53* expression (Figure 6).

## Discussion

In the present study, Naph did not influence PO_2_ crit in *C. macropomum*. The values found in this study are similar to those reported by Silva *et al.* (2019), where the mean values of PO_2_ crit were about 1.5 mg O_2_ L^-1^. The authors also did not observe differences in the PO_2_ crit of *C. macropomum* exposed to the herbicide Roundup^®^. However, Saint-Paul (1984) determined a PO_2_ crit of about 2 mg O_2_ L^-1^ for *C. macropomum.* Lower values of PO_2_ crit indicate a higher tolerance to hypoxia and guarantee the supply of oxygen to tissues (Scott *et al.*, 2008; De Boeck *et al.*, 2013; Campos *et al.*, 2016). Differences in PO_2_ crit can occur between congeneric species (Campos *et al.*, 2016), inter-individual between fish of different sizes (Scott *et al.*, 2008). This difference can also occur in fish exposed to food deprivation (De Boeck *et al.*, 2013) and different times of acclimatization to hypoxia (Regan and Richards, 2017). In the present study, data for PO_2_ crit demonstrate that intraperitoneally injected Naph did not affect oxygen consumption in *C. macropomum*.

Similarly, the absence of a Naph effect, both in normoxia and hypoxia, was reflected in hematological parameters, which were affected exclusively by the variation in oxygen concentration (Table 1). In situations where oxygen supply to tissues is impaired, one of the responses seen in fish is hematological adjustment to improve uptake and transport oxygen to tissues (Val and Almeida-Val, 1995). This response was observed only in fish exposed to hypoxia, but not in fish injected with Naph, providing support to the idea that the intraperitoneal Naph injection probably did not affect oxygen consumption in *C. macropomum*. Gesto *et al.* (2008) also did not verify changes in plasma glucose levels after Naph injection followed by acute stress stimuli in *Oncorhynchus mykiss*. Similarly, the exposure of *C. macropomum* to crude oil from the Urucu Reserve (Amazonas, Brazil) in normoxia did not result in changes in hematological parameters (Duarte *et al.*, 2010; Kochhann *et al.*, 2013).

Instead, hypoxia exposure resulted in changes in hematology (Table 1). The greater influence of oxygen concentration on hematology is supported by the PCA result, as demonstrated by the clustering of hematological variables determined by oxygen concentration (Figure 6). Hematological changes in Amazonian fish exposed to hypoxia are well reported in the literature (Val and Almeida-Val, 1995; Val, 1996; Baptista *et al.*, 2016). The increase in plasma glucose levels suggests the use of glucose as an anaerobic energy source (Chippari-Gomes *et al.*, 2005; Almeida-Val *et al.*, 2006; Baptista *et al.*, 2016). Herein, we suggest the occurrence of hepatic glycogenolysis, as demonstrated by Chippari-Gomes *et al.* (2005) in *Astronotus crassipinnis*, since the fish kept in hypoxia after the three treatments showed lower reactivity to PAS (Figure 4G and H), revealing a decrease in hepatic glycogen amount.

Only Naph injection or Naph injection followed by hypoxia influenced the biotransformation and antioxidant defenses as demonstrated by GST and CAT enzymatic activities in the present study. Other studies demonstrated that exposure of *C. macropomum* to crude oil and BaP resulted in increased GST activity (Kochhann *et al.*, 2013; Sadauskas-Henrique *et al.*, 2016). However, in the present study, *C. macropomum* injected with Naph showed a reduction in GST activity when compared to GWI and Sham in normoxia (Figure 2A). This response can be the result of an increase in hepatic lesions, such as nuclei vacuolization, which was highly frequent, possibly leading to a decrease in liver function that could not be compensated for by increased nuclei hypertrophy, which was a recurrent lesion. A decline in GST activity was also verified by Palanikumar *et al.* (2013) after exposure of *Chanos chanos* to Naph, administered directly in the water. Simultaneous exposure of *Cyprinodon variegatus* larvae to chemically enhanced water accommodated fractions (CEWAFs) and hypoxia induced the reduction in GST activity compared to the same group in normoxia (Dasgupta *et al.*, 2016). Results of the present study differ from the above described, suggesting that initial Naph injection and subsequent hypoxia exposure improved enzyme performance. Silva *et al.* (2019) also observed an increase in GST activity in *C. macropomum* contaminated with the herbicide Roundup^®^ and subsequently exposed to hypoxia, when compared to the same group in normoxia. As exposure to hypoxia increases histopathological damages, including tissue necrosis, the increase in enzyme activity may be an attempt to minimize the damage resulting from the combination of hypoxia and Naph.

CAT activity presented the same response of GST (Figure 2B). In other studies, no differences were observed in CAT activity in *C. macropomum* after exposure to crude oil and BaP (Kochhann *et al.*, 2013; Saudaskas-Henrique *et al.*, 2016). Conversely, other authors have reported a reduction in this enzyme activity after fish exposure to the water-soluble fraction of diesel oil and small Naph concentrations (Zhang *et al.*, 2004; Palanikumar *et al.*, 2013). Dasgupta *et al.* (2016) also demonstrated that exposure of *Cyprinodon variegatus* larvae to CEWAFs induced the reduction in CAT in normoxia. Some authors report on the role of hypoxia or anoxia in the activation of antioxidant defenses and the induction of oxidative stress (Lushchak *et al.*, 2001; Lushchak and Bagnyukova, 2007; Mustafa *et al.*, 2011). However, in the present study, hypoxia per se did not influence CAT activity, as observed in the GWI and Sham. In the present study, the exposure of *C. macropomum* to reduced oxygen levels did not affect the activity of the antioxidant defenses as observed in other fish species (Lushchak *et al.*, 2001; Lushchak and Bagnyukova, 2007). However, the Naph injection with subsequent hypoxia exposure appears to have improved CAT performance. Similar to what happened with GST activity, this increase in CAT activity may be a way to minimize the damage caused by exposure to Naph plus hypoxia. Silva *et al.* (2019) demonstrated an increase in CAT activity in fish exposed to Roundup^®^ and subsequently exposed to hypoxia, which was accompanied by increased histopathological damage in the liver.

No differences were observed in liver LPO in *C. macropomum* between treatments in both fish groups under normoxia or hypoxia, contrasting with the results of Palanikumar *et al.* (2013), who observed changes in LPO concentration after exposure of *Chanos chanos* to Naph, administered directly in water. However, Kochhann *et al.* (2013) did not find differences in lipid peroxidation in *C. macropomum* exposed to crude oil and neither did Dasgupta *et al.* (2016) observe variation in LPO after the simultaneous exposure of *Cyprinodon variegatus* larvae to CEWAFs plus normoxia or hypoxia. Silva *et al.* (2019) verified that there was no difference in the LPO in C*. macropomum* under hypoxic conditions, and in fish exposed to the herbicide Roundup^®^ plus hypoxia there was a reduction in LPO.

Fish contaminated with Naph showed an increase in the DNA damage index in blood cells when kept in normoxia or exposed to hypoxia (Figure 3). Other studies have reported similar genetic damage in fish exposed to crude oil and PAHs, without, however, evaluating exposure to abiotic factors such as hypoxia (Kochhann *et al.*, 2013; Palanikumar *et al.*, 2013; Sadauskas-Henrique *et al.*, 2016; Silva *et al.*, 2017). The observed DNA damage in the present study caused by Naph in fish kept under normal oxygen conditions may be the result of the biotransformation process and antioxidant defenses that were inefficient, as demonstrated by the inhibition of the GST and CAT enzymatic activities, which have contributed to the increase of class 2 damage. Previous studies support this idea, as they show that the genotoxicity of Naph results from the inhibition of detoxification mechanisms (Teles *et al.*, 2003; Palanikumar *et al.*, 2013).

Hypoxia, independently, did not induce genotoxic damage in *C. macropomum* blood cells, which was corroborated by PCA, differing from previous studies (Mustafa *et al.*, 2011; Negreiros *et al.*, 2011). Just as we observed herein, Dasgupta *et al.* (2016) also demonstrated that hypoxia per se did not induce DNA damage in larvae of *Cyprinodon variegatus*. However, hypoxia is considered a factor that enhances DNA damage in fish exposed to both chemical and physical stressors (Groff *et al.*, 2010; Mustafa *et al.*, 2011, 2012; Negreiros *et al.*, 2011; Dasgupta *et al.*, 2016; Silva *et al.*, 2019). In the present study, hypoxia exposure did not magnify genotoxic damages in fish previously contaminated with Naph, and the observed injuries were caused exclusively by Naph (Figure 3). Silva *et al.* (2019) also demonstrated that the exposure of *C. macropomum* to Roundup^®^ and subsequent submission to hypoxia did not affect or magnify DNA damages. However, the hisptopatological response was different.

Many authors report histopathological liver damages in fish exposed to crude oil or derivatives (Akaishi *et al.*, 2004; Gusmão *et al.*, 2012; Silva *et al.*, 2017). Herein, most of the alterations showed low to moderate frequencies (Table 2). Naph injection in normoxia resulted, as expected, increased nuclei hypertrophy, cell deformation, and nuclei vacuolation frequencies (Figure 4B, C). Exposure of *C. macropomum* to BaP via intraperitoneal injection also resulted in hepatic lesions that were even more severe than those observed in the present study (Silva *et al.*, 2017), perhaps because BaP is considered to be more toxic than Naph. Liver histopathological damage was reported for *Astyanax* sp. (Akaishi *et al.*, 2004) and *Odontesthes argentinensis* (Gusmão *et al.*, 2012) exposed to the water-soluble fraction of crude oil. Omar-Ali *et al.* (2015) also demonstrated the occurrence of hepatic lesions in *Atractosteus spatula* exposed to the water-accommodated fraction of crude oil. In the present work, subsequent hypoxia exposure also induced a higher intensity in liver histopathological damages (Figure 4D and E) in all treatments. Although necrotic foci were present in all treatments, their frequency was low and punctually localized, except for fish contaminated with Naph and subsequently exposed to hypoxia, where necrosis was frequent and widely distributed, causing disorder in the analyzed tissue (Figure 4E). Silva *et al.* (2019) demonstrated that hypoxia induced and accentuated hepatic lesions in *C. macropomum* after exposure to the herbicide Roundup^®^, increasing, mainly, foci of necrosis. Mustafa *et al.* (2012) also reported that hypoxia induced hepatic damage in *Cyprinus carpio* after simultaneous exposure to copper. In this wok, the hepatic lesions occurred in fish exposed to Naph plus hypoxia, even without increasing LPO content, which was also observed by Silva *et al.* (2019). Due to the increase in liver damage of fish exposed to Naph and hypoxia, there is an attempt to minimize the negative effects of the combination of the stressors, through an increase in GST and CAT activity, as observed in this work.

Regarding the expression of the tumor suppressor gene *tp53* in fish from the Naph group in both normoxic and hypoxic conditions (Figure 5), the increase in gene expression was probably related to the histopathological damages resulting from Naph exposure, thus avoiding that the negative effects of Naph be passed on. Naph, as well as other PAHs, are considered genotoxic, promoting DNA damage in different tissues, such as liver, gills, and blood (Teles *et al.*, 2003; Palanikumar *et al.*, 2013, Sadaukas-Henrique *et al.*, 2017). Increased p53 protein levels after exposure to DNA damaging agents have been reported for mammals (Kastan *et al.*, 1991; Park *et al.*, 2006). In fish, genotoxic xenobiotics exposure is accompanied by an increase in *tp53* mRNA levels (Mai *et al.*, 2012). Increased histopathological damage, observed in this study, might have modulated the expression of *tp53*, inducing a cell cycle block that prevents possible damage from being passed on to new cells, as an attempt to avoid aggravation of the hepatic lesion. A study by Tu *et al.* (2016) demonstrated that the exposure of the *tp53* null mutant strain *Oryzias latipes* to propiconazole resulted in increased hepatic lesions when compared to wild-type fish, presenting even greater susceptibility to hepatocarcinogenesis. An increase in *tp53* transcripts was also observed in fish exposed to heavy fuel oil (Ruiz *et al.*, 2012) and in fish captured at contaminated sites (Williams and Hubberstey, 2014), without, however, considering the effect of hypoxia exposure.

Hypoxia is also a factor related to the regulation of the gene *tp53*. However, studies on the role of hypoxia in the regulation of this gene have been mostly performed in mammalian cells under *in vitro* conditions, and these report controversial results, such as accumulation (Koumenis *et al.*, 2001; Hammond *et al.*, 2002), or decrease in p53 protein levels (Cosse *et al.*, 2009; Chen *et al.*, 2010). Studies on the effects of hypoxia on *tp53* gene expression in fish are scarce to date and, for the best of our knowledge, this is the first study that evaluates this gene expression in an Amazonian fish species under the effects of hypoxia after a PAH injection. The role of hypoxia in *tp53* gene regulation in the present study was more intense than that of Naph injection, as shown by the F values for oxygen concentration (53.935) and treatments (34.470) and after the PCA analysis (Figure 6). Although hypoxia induced the decrease of *tp53* transcripts in all treatments, in fish injected with naphthalene there was a clear attempt to maintain the response similar to group in normoxia, with increased transcription of the gene. A similar response, to minimize damage resulting from the combination of Naph and hypoxia was also observed in the activity of the GST and CAT enzymes. However, in fish treated with Naph in normoxia, this increase in the number of transcripts was about 2 fold greater than in the same group in hypoxia. The lower efficiency to activate *tp53* gene transcription could have occurred due to the combination between increased nuclei vacuolation and focal necrosis intensity, which may have resulted in the reduction of functional hepatocytes, thus compromising organ function.

In mammalian cells, hypoxia exposure did not change *tp53* mRNA levels (Koumenis *et al.*, 2001; Chen *et al.*, 2010), differing from the results of the present study, indicating that the destruction of tissue and effects on hepatocytes were more severe when we used fish as model and *in vivo* experiments. These responses suggest that, unlike the previously proposed genotoxic effects for contamination with xenobiotics such as naphthalene, hypoxia promotes a differential regulation of *tp53* expression, and differs between animal models (mammals) and experimental biological types (whole live fish and isolated live cells). Recent studies with white shrimp (*Litopenaeus vannamei*) have shown that hypoxia affects the transcription of *tp53*. Nuñez-Hernandez *et al.* (2018) demonstrated that exposure of *L. vannamei* to 48 h of hypoxia resulted in increased *tp53* transcript levels in the hepatopancreas. Controversial results have been reported by Felix-Portillo *et al.* (2016), who demonstrated that 48 h of exposure to hypoxia resulted in a reduction in *tp53* transcripts in hemocytes. Time of exposure to hypoxia may explain these diferences, since hypoxia induces metabolic depression in some animals (Almeida-Val *et al.*, 2000).

Further studies need to be performed for a better understanding of the effects of hypoxia on the regulation of *tp53* gene expression and p53 protein levels in fish. Moreover, one should consider that the *in vivo* responses of the organism to hypoxia are much more complex than the responses of cells *in vitro*, particularly when the model is Amazonian fish that, due to the seasonal and daily variations in the dissolved oxygen concentration, have developed adaptations to survive long and intermittent periods of oxygen shortage (Val and Almeida-Val, 1995). The decrease in *tp53* gene expression in GWI and Sham after hypoxia exposure might be related to metabolic depression plus the increased frequency of hepatic lesions. Nuclei vacuolization is considered a sign of a degenerative process that reduces metabolically active areas of the liver, resulting in a decrease in hepatic functions (Pacheco and Santos, 2002; Camargo and Martinez, 2007; Benze *et al.*, 2014). Fish under these circumstances may not be able to compensate for these lesions, even though there was a significant occurrence of nuclei hypertrophy, demonstrating a tentative increase in cell activity (Benze *et al.*, 2014).

## Conclusion

Hypoxia accentuated the harmful effects of naphthalene injection, with the exception of hematological and genotoxicity (comet assay) parameters. Naph and hypoxia had an opposite effect on *tp53* tumor suppressor gene regulation. The *tp53* down-regulation seen in hypoxic fish differs from that occurring in mammalian cells and, in the present study, it might be related to metabolic depression mechanisms in these fish, plus the observed higher intensity of hepatic lesions. For a better comparison of studies between mammalian and fish cells, hepatocyte cultures should be used for comparative analyses at the p53 protein level. The increase in *tp53* mRNA levels, as well as the increase in GST and CAT activities in fish treated with Naph and subsequently exposed to hypoxia, provides clear evidence that, even under low oxygen conditions, individuals of *C. macropomum,* injected with genotoxic agents, invest in the defense of liver cells. Thus, given the aquatic contamination, this species increased, preferentially, the *tp53* gene transcription, as an attempt to ensure the maintenance of genomic integrity, but this was not as efficient as in fish kept in normoxia, demonstrating that responses at the transcriptional level of the *tp53* gene in fish may be compromised by hypoxia. This response reflects the importance of considering hypoxia as an additional risk factor for aquatic contamination, especially in an environment where hypoxic conditions occur both daily and seasonally, besides being a parameter that contributes to the development and greater severity of cancer.
